# BK channel blocker paxilline attenuates thalidomide-caused synaptic and cognitive dysfunctions in mice

**DOI:** 10.1038/s41598-018-36367-3

**Published:** 2018-12-05

**Authors:** Tae-Yong Choi, Seung-Hyun Lee, Soo-Jeong Kim, Youhwa Jo, Chul-Seung Park, Se-Young Choi

**Affiliations:** 1 Department of Physiology and Dental Research Institute, Seoul National University School of Dentistry, Seoul, 03080 Republic of Korea; 2 School of Life Sciences, Gwangju Institute Science and Technology, Gwangju, 61005 Republic of Korea; 3 Department of Neural Development and Disease, Korea Brain Research Institute, Daegu, 41068 Republic of Korea

## Abstract

Thalidomide is a widely prescribed immunomodulatory drug (iMiD) for multiple myeloma, but causes reversible memory loss in humans. However, how thalidomide causes cognitive dysfunction at a cellular and molecular level has not been demonstrated. We studied the effect of thalidomide on synaptic functions and cognitive behaviors using a mouse model. Thalidomide led to cognitive deficits in learning behavior in a passive avoidance test and in a novel object recognition test, increased anxiety in an elevated plus maze test, and increased depressive behaviors in a tail suspension test. Interestingly, thalidomide elevated big- or large-conductance, calcium-activated K^+^ (BK) channel expression in the plasma membrane and BK channel activity in the hippocampus. Thalidomide also increased the paired pulse ratio of excitatory postsynaptic current (EPSC), which suggests a decreased probability of glutamate release. Furthermore, the changes in the paired pulse ratio and in BK channel activity were blocked by paxilline, a BK channel blocker. Finally, we found that thalidomide-induced cognitive dysfunctions were restored by paxilline treatment. These results suggest that thalidomide-mediated BK channel hyperfunction is responsible for the pathological mechanism of thalidomide-associated reversible memory loss.

## Introduction

Thalidomide, which was developed as a sedative but was subsequently determined to be a teratogen that affects limb formation, has recently been re-evaluated as an immunomodulatory drug^1,2^. Thalidomide and its derivatives (such as lenalidomide and pomalidomide) are widely prescribed for multiple myeloma, myelodysplastic syndrome, and autoimmune diseases^3,4^. However, thalidomide and its derivatives produce a side effect of reversible memory loss^5,6^. The cognitive impairment side effect of these immunomodulators is important because they can also influence drug selection^7^. Memory loss due to chemotherapy, called chemotherapy-induced cognitive impairment, is a very common problem in drug choice^8^.

Thalidomide acts on *cereblon* (*Crbn*), a gene on human chromosome 3p26.3 that causes intellectual disability in humans when mutated, and leads to inhibition of CRL4^CRBN^ E3 ubiquitin ligase-related functions^9–14^. Thalidomide affects many targets through CRL4^CRBN^ E3 ubiquitin ligase^15–17^. For instance, thalidomide-induced limb malformation has been found to be related to CRL4^CRBN^-dependent ubiquitination of fibroblast growth factor Fgf8^18^. Thalidomide also causes the ubiquitination of casein kinase 1A1 (CK1α), which eventually induces degradation of CK1α^19,20^ and/or GSPT1^21^. In addition, E3 ubiquitin ligase-independent mechanisms have also been reported: thalidomide interferes with the cereblon-CD147-MCT1 transmembrane complex, which results in pleiotropic antitumor activity and teratogenicity^22^. However, the mechanism of thalidomide-mediated reversible memory loss is not yet understood. Elucidating the mechanism is important from the perspective of not only understanding chemotherapy-induced cognitive impairment, but also for manipulating the therapeutic efficacy of thalidomide.

Recently, the mechanism of cognitive impairment due to a loss-of-function mutation in CRBN, a molecular target of thalidomide, has been elucidated^23–25^. These findings provided clues that are important for tracking the mechanism of action of thalidomide. In this report, we characterize cognitive dysfunction in thalidomide-treated mice and identify effects of thalidomide on BK channel activity, one of the thalidomide-CRBN functional targets.

## Results

### Thalidomide treatment induced abnormal cognitive behaviors

**Figure 1:**
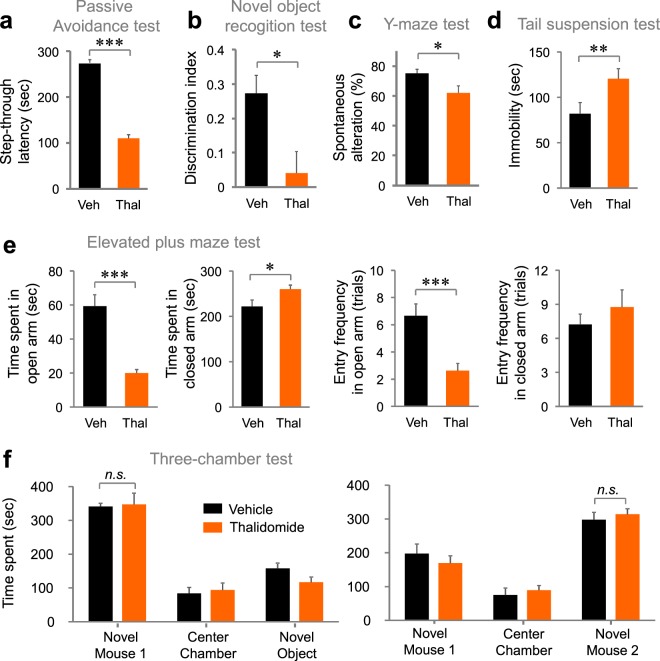
Thalidomide treatment induces behavioral abnormalities in mice. (**a**) Thalidomide-treated mice (Thal, 30 mg/kg, i.p.) spent significantly less time enter to the dark chamber on day 2 of the passive avoidance test than did vehicle-treated control mice (Veh). n = 7 (vehicle); n = 6 (thalidomide). (**b**) Thalidomide-treated mice spent less time exploring the novel object than the familiar object in the novel object recognition test. n = 5 (vehicle); n = 6 (thalidomide). (**c**) Thalidomide-treated mice showed decreased spontaneous alterations in the Y-maze test. n = 7 (vehicle); n = 8 (thalidomide). (**d**) Thalidomide-treated mice showed increased immobility time in the tail-suspension test. n = 9 (vehicle); n = 8 (thalidomide). (**e**) Thalidomide-treated mice showed decreased time and entry trial in the open arms and an increased time and entry trial in the closed arms of the elevated plus maze test. n = 9 (vehicle); n = 8 (thalidomide). (**f**) No differences were observed between vehicle-treated and thalidomide-treated mice in the three-chamber social interaction test. n = 8 (vehicle); n = 7 (thalidomide). *P < 0.05; **P < 0.01; ***P < 0.001; n.s., not significant (by unpaired Student’s t-test).

We tested thalidomide-induced cognitive dysfunction in mice similar to that reported for human patients^6^. We first tested whether thalidomide treatment mimicked the behavioral changes of *Crbn* KO mice that we and other groups had recently reported^23–26^. We found that thalidomide-treated mice showed a decrease in step-through latency in the passive avoidance test (Fig. 1a, t(11) = 17.013, *P* < 0.001 by unpaired Student’s t-test). Thalidomide-treated mice spent significantly less time exploring the novel object than exploring the familiar object (Fig. 1b, t(9) = 2.767, *P* = 0.0219 by unpaired Student’s t-test). Thalidomide also decreased spontaneous alterations in Y-maze tests (Fig. 1c, t(15) = 2.233, *P* = 0.0437 by unpaired Student’s t-test). These results suggest that thalidomide aggravates cognitive function in naïve animals similarly to what has been observed in human multiple myeloma patients who were prescribed IMiD. Thalidomide-treated mice also showed more immobility in a tail suspension test (Fig. 1d, t(17) = −3.198, *P* = 0.00527 by unpaired Student’s t-test), and, in the elevated plus maze test, mice spent less time and decreased entry trials in the open arms (Fig. 1e, t(15) = 7.159, *P* < 0.001 (time spent in open arms); t(15) = −2.908, *P* = 0.0108 (time spent in closed arms); t(15) = 5.101, *P* < 0.001 (entry frequencies in open arms); t(15) = −1.135, *P* = 0.274 (entry frequencies in closed arms) by unpaired Student’s t-test). These results indicate that thalidomide can generate or exacerbate neuropsychiatric disorders, such as anxiety or depression. However, thalidomide-treated mice showed normal social preference and social novelty recognition of novel mice in the three-chamber test (Fig. 1f, t(13) = −0.204, *P* = 0.421 (social preference); t(13) = −0.702, *P* = 0.247 (social novelty) by unpaired Student’s t-test).

### Thalidomide treatment induced BK channel hyperactivity

**Figure 2:**
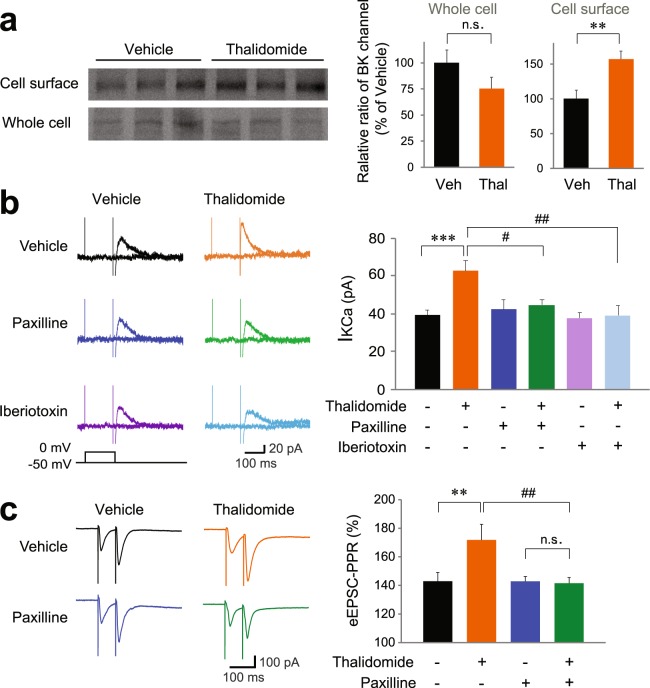
Thalidomide treatment increases BK channel activity and reduces presynaptic glutamate release probability. (**a**) Surface expression of BK channels increased in thalidomide-treated hippocampal slices. Hippocampal slices prepared from vehicle (Veh) and thalidomide (Thal)-treated mice were used for steady-state biotinylation of surface BK channels. Input (25%) of total lysates are shown in the bottom panel, and the biotinylated surface BK channels are shown in the top panel. The quantification of biotinylated BK channels and total BK channels is presented as mean ± SEM. n = 6. (**b**) The increase in calcium-activated potassium currents (I_K(Ca)_) in hippocampal CA1 pyramidal neurons due to a 3 h incubation with 100 μM thalidomide was reduced by bath-application of 10 μM paxilline or 100 nM iberiotoxin. I_K(Ca)_ was evoked by brief depolarization from the holding potential (−50 mV) under a TTX-including external solution. n = 32 slices, 9 mice (vehicle); n = 42, 7 (thalidomide); n = 17, 5 (paxilline); n = 19, 5 (thalidomide + paxilline); n = 12, 4 (iberiotoxin); n = 12, 4 (thalidomide + iberiotoxin). (**c**) Paxilline (10 μM) abolishes the increased paired-pulse ratio (PPR, 50 ms inter stimulus interval) at SC-CA1 synapses in mouse hippocampal slices initially incubated with 100 μM thalidomide for at least 3 h. n = 22, 5 (vehicle); n = 19, 6 (thalidomide); n = 29, 4 (paxilline); n = 20, 3 (thalidomide + paxilline). ^*#*^*P* < 0.05; **P or ^*##*^*P* < 0.01; ***P  < 0.001; n.s., not significant. (*Asterisk: unpaired Student’s t-test or two-way ANOVA, ^#^Sharp: Post-hoc multiple comparison test).

It has been reported that CRBN interacts with BK channels^27^ and *Crbn* KO mice had more BK channels exposed to the plasma membrane, and BK channel activity was increased^25^. We examined whether these results from the *Crbn* KO mice were also observed after thalidomide treatment. To test this, we biotinylated proteins in the plasma membrane and quantified biotinylated proteins versus total proteins. Interestingly, thalidomide treatment slightly increased the amount of biotinylated (surface) BK channels (Fig. 2a). Next, we tested the effect of thalidomide on synaptic function. Thalidomide significantly increased Ca^2+^-activated K^+^ currents (I_K(Ca)_) in hippocampal CA1 pyramidal neurons. The increase was blocked by 10 μM paxilline, a BK channel inhibitor (Fig. 2b, F(1,106) = 5.450, *P* = 0.021 (thalidomide); F(1,106) = 1.857, *P* = 0.176 (paxilline); F(1,106) = 3.828, *P* = 0.017 (thalidomide + paxilline) by two-way ANOVA with Holm-Sidak multiple comparison test; *P* < 0.001 (control vs. thalidomide), *P* = 0.016 (thalidomide vs. thalidomide + paxilline)). We confirmed the inhibition of BK channel activity with 100 nM iberiotoxin, another BK channel blocker (Fig. 2b, F(1,94) = 3.673, *P* = 0.058 (thalidomide); F(1,94) = 3.847, *P* = 0.053 (iberiotoxin); F(1,94) = 2.969, *P* = 0.088 (thalidomide + iberiotoxin) by two-way ANOVA with Holm-Sidak multiple comparison test; *P* = 0.009 (thalidomide vs. thalidomide + iberiotoxin)). To monitor the effects of thalidomide on presynaptic release, we analyzed the paired pulse ratio (PPR) using two separate stimulations. Interestingly, preincubation with 100 μM thalidomide increased the PPR in EPSC evoked by the stimulation of hippocampus Schaffer collateral (SC)-CA1 circuit. In addition, the thalidomide-mediated increase in PPR was blocked by paxilline (Fig. 2c, F(1,86) = 4.925, *P* = 0.029 (thalidomide treatment); F(1,86) = 5.984, *P* = 0.016 (paxilline treatment); F(1,86) = 5.924, *P* = 0.017 (Interaction) by two-way ANOVA with Holm-Sidak multiple comparison test; *P* = 0.002 (control vs. thalidomide), *P* = 0.002 (thalidomide vs. thalidomide + paxilline)). These results suggest that the BK channel hyperactivity that had been shown in *Crbn* KO mice is also caused by thalidomide.

### BK channel blockers rescue thalidomide-induced changes in cognitive function

**Figure 3:**
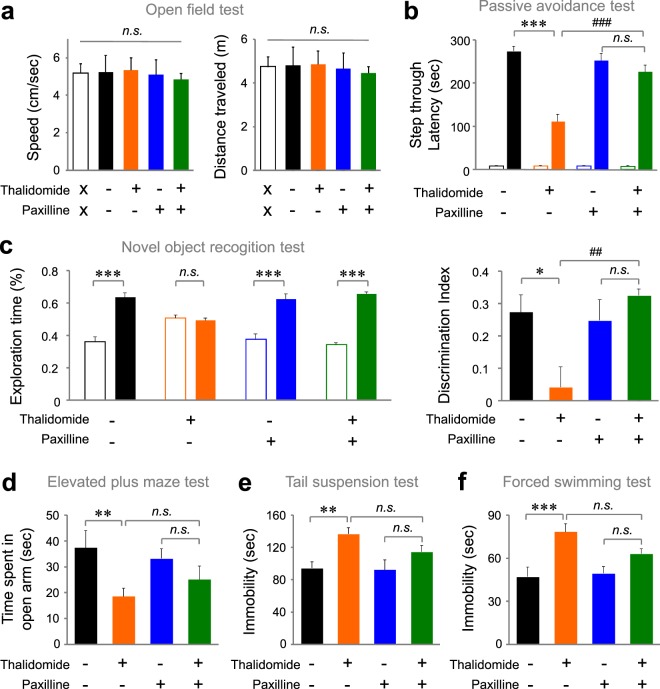
Paxilline rescues the thalidomide-mediated alterations in cognitive functions. (**a**) Thalidomide (30 mg/kg, i.p.) and/or paxilline-treated mice (3 μg/kg, i.p.) show normal locomotor activity. (left) Speed, (right) Distance traveled. n = 5 (Saline, Blank); n = 7 (vehicle); n = 8 (thalidomide); n = 8 (paxilline); n = 8 (thalidomide + paxilline). Blank bar depicts the untreated control without any chemical or vehicle. (**b)** Thalidomide-treated mice spent significantly less time enter to the dark chamber on day 2 of the passive avoidance test, which was reversed by paxilline treatment. n = 20 (vehicle); n = 19 (thalidomide); n = 19 (paxilline); n = 20 (thalidomide + paxilline). (**c**) Paxilline treatment rescued the abnormal behavior of thalidomide-treated mice in the novel object recognition test. (Left) exploration time, (right) discrimination index. n = 5 (vehicle); n = 6 (thalidomide); n = 5 (paxilline); n = 6 (thalidomide + paxilline). (**d**) Decreased time spent in open arms of the EPM test was partially reversed by paxilline treatment. n = 7 (vehicle); n = 8 (thalidomide); n = 7 (paxilline); n = 7 (thalidomide + paxilline). (**e**,**f)** The increase in immobility time by thalidomide treatment was restored by paxilline. (**e**) Tail-suspension test, n = 7 (vehicle); n = 7 (thalidomide); n = 7 (paxilline); n = 6 (thalidomide + paxilline). (**f**) Forced-swim test, n = 7 (vehicle); n = 8 (thalidomide); n = 7 (paxilline); n = 7 (thalidomide + paxilline). *P < 0.05; **P or ^*##*^*P* < 0.01; ***P or ^*###*^*P* < 0.001; n.s., not significant. (*Asterisk: unpaired Student’s t-test or two-way ANOVA, ^#^Sharp: Post-hoc multiple comparison test).

We next tested whether paxilline rescues thalidomide-mediated impaired cognitive function as well as altered presynaptic release. In the open field test, thalidomide-injected mice showed normal locomotor activity and paxilline did not affect the basal locomotion (Fig. 3a; Locomotive speed, F (1,27) = 0.67, P = 0.41 (Thalidomide treatment); F = 0.02, P = 0.88 (paxilline treatment); F = 0.14, P = 0.71 (interaction); Distance traveled, F(1,27) = 0.64, P = 0.43 (thalidomide treatment); F = 0.03, P = 0.85 (paxilline treatment); F = 0.13, P = 0.71 (interaction) by two-way ANOVA). Treatment with paxilline restored thalidomide-mediated decreased step-through latency in the passive avoidance test (Fig. 3b, F(1,74) = 38.975, *P* < 0.001 (thalidomide); F(1,74) = 9.616, *P* = 0.003 (paxilline); F(1,74) = 20.456, *P* < 0.001 (Interaction) by two-way ANOVA with Bonferroni’s multiple comparison test, *P* < 0.001 (control vs thalidomide), *P* < 0.001 (thalidomide vs thalidomide + paxilline)). We also confirmed that thalidomide-mediated changes in exploration time and the reduction in the discrimination index were rescued by paxilline in the novel object recognition test (Fig. 3c; Exploration time, t(8) = 7.32, P < 0.001 (vehicle); t(10) = 0.93, P = 0.37 (thalidomide); t(8) = 5.33, P = 0.0007 (paxilline); t(10) = 21.65, P < 0.001 (thalidomide + Paxilline) by unpaired Student’s t-test; Discrimination index, F(1,18) = 5.95, *P* = 0.025 (thalidomide); F(1,18) = 2.16, *P* = 0.159 (paxilline); F(1,18) = 8.66, *P* = 0.008 (Interaction) by two-way ANOVA with Bonferroni’s multiple comparison test, *P* < 0.05 (control vs thalidomide), *P* < 0.01 (thalidomide vs thalidomide + paxilline)). Paxilline also partially rescued other behavioral changes induced by thalidomide treatment. In the elevated plus maze test, paxilline normalized the increased time spent in open arm in thalidomide-treated mice (Fig. 3d; F(1,25) = 7.80, *P* < 0.01 (Thalidomide); F(1,25) = 0.06, *P* = 0.80 (paxilline); F(1,25) = 0.23, *P* = 0.27 (interaction) with Bonferroni’s multiple comparison test, *P* < 0.01 (control vs thalidomide), *P* > 0.05 (thalidomide vs thalidomide + paxilline)). Morevoer, paxilline normalized the increased immobility time in tail-suspension test (Fig. 3e; F(1,23) = 11.81, *P* < 0.01 (Thalidomide); F(1,23) = 1.69, *P* = 0.20 (Paxilline); F(1,23) = 1.13, P = 0.29 (interaction) with Bonferroni’s multiple comparison test, *p* < 0.01 (control vs thalidomide), *P* > 0.05 (thalidomide vs thalidomide + paxilline)) and in forced-swimming test (Fig. 3f; F(1,25) = 18.25, *P* < 0.001 (Thalidomide); F(1,25) = 1.47, *P* = 0.23 (Paxilline); F(1,25) = 2.76, *P* = 0.10 (interaction) by two-way ANOVA with Bonferroni’s multiple comparison test, *P* < 0.001 (control vs thalidomide), *P* > 0.05 (thalidomide vs thalidomide + paxilline)). Therefore, we conclude that the inhibition of CRBN activity by thalidomide causes synaptic and behavioral changes via upregulation of BK channel activity.

## Discussion

In this study, we investigated the causal mechanism of cognitive impairment by thalidomide. First, we examined whether thalidomide treatment of a mouse model mimics the cognitive dysfunctions observed in human patients. Thalidomide induced the impairment of spatial association in the passive avoidance test (Fig. 1a), reduced recognition memory and decreased preference for novel objects (Fig. 1b), and inhibited working memory in the Y-maze test (Fig. 1c). Thalidomide-treated mice showed depressive behavior in the tail-suspension test (Fig. 1d) and hyper-anxiety in the elevated plus maze test (Fig. 1e). However, thalidomide did not affect social activity (Fig. 1f). These results suggest that thalidomide causes cognitive impairment in the mouse model.

Next, we examined the effect of thalidomide on synaptic function in the hippocampus. Thalidomide increased Ca^2+^ activated K^+^ channel current. Since paxilline blocks the increased portion of K^+^ current, the increased K^+^ current is primarily mediated by BK channel activity. In general, an increase in BK channel activity affects action potential duration and reduces presynaptic glutamate release. Recently RimBP2 is reported to localize BK channel in the presynaptic terminal and enables proper presynaptic release functions^28^. In our results, thalidomide increased PPR of the EPSC, indicating a decrease in the probability of glutamate release in hippocampal SC-CA1 circuits. Interestingly, this effect was abolished by paxilline, which suggests that thalidomide induces hyperfunction of BK channels and therefore reduces the probability of presynaptic neurotransmitter release. We confirmed that paxilline also rescues thalidomide-mediated impairment of cognitive functions in the passive avoidance and novel object recognition tests. These results indicate that thalidomide-induced cognitive dysfunction is due to BK channel hyperfunction. Our study suggests that BK channel hyperfunction is the cellular and molecular mechanism underlying the memory loss side effect of thalidomide.

Recently, the molecular mechanisms of CRBN-related intellectual disability have been elucidated^23–25^. We had shown that *Crbn KO* animals with cognitive deficits showed normal synaptic morphology and long-term plasticity, but exhibited increased dysfunction of BK channel activity-mediated presynaptic glutamate release^25^. However, in these reports, the phenotypes of the *Crbn* KO animals had slightly differed from each other. We believe this can be explained as follows. First, the mechanism of CRBN in different neurons is not the same. Interestingly, the spatial memory defect in *Crbn* KO mice was reversed by the BK channel blocker, whereas the hyperanxious behavior in KO mice was not reversed by the BK channel blocker^25^. In addition, CRBN and its target can vary by brain area and neuronal circuit. The hyperanxious behavior of *Crbn* KO mice was not observed in the alpha-CaMK-conditioned *Crbn KO* animals. These behavioral abnormalities were attributable to CRBN abnormalities in the brain region independent of the hippocampus forebrain region^26^. In other words, not all behavioral phenotypes in *Crbn* KO mice are BK channel- and forebrain-dependent. However, since the phenotypes of *Crbn* KO mice related to at least intellectual disability were BK channel dependent, we hypothesized that thalidomide causes reversible dementia through a mechanism similar to the mechanism in *Crbn* KO mice. Our results showed that thalidomide increased BK channel activity and mimicked the synaptic and behavioral phenotypes of *Crbn* KO mice. We also found that a pharmacological rescue experiment with a BK channel blocker recovered synaptic and behavioral dysfunction in thalidomide-treated mice. Recently, the K^+^ channel has attracted attention as an important modulator of cognitive function. Our findings suggest that synaptophysiology and channelopathy are molecular mechanisms of thalidomide-mediated cognitive impairment.

Interestingly, there seemed to be a discrepancy between the levels of surface expression and the activity of BK channel. Our results show that BK channel activity was rarely observed in normal condition (Fig. 2b), although the surface expression of BK channel was readily detected (Fig. 2a). The underlying reason for the discrepancy is unclear but several explanations might be possible: (1) thalidomide affects factors that directly or indirectly modulate BK channel activity, (2) thalidomide affects synaptic proteins involved in the localization or co-localization of the BK channel and the voltage-sensitive Ca^2+^ channel (VSCCs), which increases Ca^2+^ concentration that can enhance the activity of the BK channel, (3) thalidomide selectively modulates the localization of the BK channel to the axon terminal (where VSCCs are enriched), and/or (4) thalidomide affects the surface expression of VSCC. Further experiments are needed to provide a clear mechanism.

Recently, there have been attempts to use thalidomide for the treatment of Alzheimer’s disease and brain injury. Thalidomide has been reported to suppress memory loss caused by chronic neuroinflammation^29^ and sleep deprivation^30^. Therefore, thalidomide appears to have a neuroprotective effect on pathological conditions independent of memory loss itself. This may be attributed to the various targets of thalidomide, and further analysis of these effects will be an important topic in understanding the effects of thalidomide.

Taken together, we have found that thalidomide induces the hyperfunction of BK channels and reduces the probability of presynaptic glutamate release. We also confirmed that paxilline, a BK channel blocker, restored synaptic and cognitive impairment caused by thalidomide. We expect that our results will contribute to increasing the utility of thalidomide by providing a better understanding of the causal mechanisms underlying its side effects and mitigating those side effects.

## Materials and Methods

### Experimental animals

C57BL6 male mice were 2–4 weeks old for the electrophysiological experiments and 7–11 weeks old for the behavioral experiments. All mice were purchased from DBL (Eumseong, South Korea) and were housed in an animal facility with a specific pathogen-free barrier under a 12-h light/dark cycle. Mice were allowed access to food and water *ad libitum*. All experiments were approved by the Institutional Animal Care and Use Committee (IACUC) at Seoul National University. We also confirm that all methods were performed in accordance with the relevant guidelines and regulations of Seoul National University and Korean government (Ministry of Science and ICT).

### Behavior tests

#### Open field test

Spontaneous exploratory activity were assessed in an automated open field. The open field test as performed as previously described^31^. In brief, mice were placed in a acryl box (45 W × 45 D × 30 H cm^3^), and mouse movements were recorded and analyzed by real-time video-tracking computer software Smart Version 2.5 (Panlab-Harvard Apparatus) linked to an overhead video camera. The total distance traveled was measured for 15 min. The open field arena was cleaned with 70% ethanol and wiped with paper towels after each trial.

#### Passive avoidance test

The test was performed as described previously^25,31^. The observer was blinded to the pharmacological treatment of the animal. The passive avoidance test apparatus consisted of a light and dark chamber separated by a retractable door. The floor of the dark chamber was made of stainless-steel grids. During habituation, mice were allowed to freely explore the box for 5 min with the door open, and were then returned to their home cage. For conditioning, after 24 h, the mice were placed into the light chamber, and the sliding door was closed when both hindlimbs of the mouse had entered into the dark chamber. Then, an electric foot shock (0.25 mA, 2 s) was delivered via the floor grids. Ten seconds later, the mice were returned to their home cage. Tests were carried out 24 h after conditioning, and the latency time required for mice to enter the dark chamber was measured with a 300-second cut-off.

#### Tail-suspension test

Mice were suspended head-down by their tails with adhesive tape on a bracket 50 cm from the ground. Immobility time was recorded with a stopwatch over a 5-min test period.

#### Forced swimming test

The test was performed by using a standard protocol of forced-swim test for mice. Briefly, a transparent acrylic beaker (10 cm in diameter, 20 cm height) filled with water (23–25 °C) to a depth of 16 cm was used as the apparatus. Mice were placed in the beaker and allowed to swim undisturbed for 6 min and then removed, dried, and returned to their home cages. Water was changed between each subject. Immobility time was recorded with a stopwatch, during the last 4 min of the test session.

#### Elevated plus maze test

The elevated plus maze apparatus was a plus-shaped maze elevated 60 cm above the floor. It consisted of two closed arms surrounded by 30 cm high opaque walls and two open arms (110 × 110 cm). Each mouse was placed in the center (5 × 5 cm) of the maze facing one of the closed arms and allowed to explore the space for 15 min. The movements of the mice were tracked and recorded via an overhead video camera and analyzed by real-time video-tracking computer software, Smart Version 2.5 (Panlab-Harvard Apparatus, Holliston, MS, USA). Time spent in the closed arms, center, and open arms was measured. The maze was cleaned with 70% ethanol and wiped with paper towels between each trial.

#### Three-chamber social interaction test

This test was performed as described previously with minor modifications^31,32^. A three-chamber apparatus was made using transparent Plexiglas (60 [W] × 45 (D) × 40 [H] cm^3^) with two transparent partitions dividing the left, center, and right chambers (20 × 45 cm, each). Each partition had a square opening (10 × 10 cm) in the bottom center. A cylindrical wire cage (8.5 cm diameter) was used as an inanimate object. A cylindrical bottle filled with water was placed on the top of the wire cup to prevent the mouse from climbing to the top of the cup. The three-chamber unit and wire cups were cleaned with 70% ethanol and wiped with paper towels between each trial. The three-chamber test was composed of three phases. First, mice were habituated in the three-chamber apparatus for 10 min. In the second phase, an age- and gender-matched mouse of the same strain that had never been exposed to the test mouse (M1) was placed in one of the two wire cages. The empty wire cage served as an inanimate object (O) cue and was placed on the other side. Then, the test mouse was placed in the center and allowed to freely explore the chamber for 10 min. In the third phase, a second age- and gender-matched mouse (M2) that had never been exposed to the test mouse was placed in the previously empty wire cage. Thus, the test mouse would have a choice between a familiar mouse (M1) or a novel mouse (M2). The test mouse was placed in the center and allowed to freely explore the chamber for 10 min. Exploration was defined as each instance in which the test mouse sniffed the empty cage/mouse or oriented its nose toward and came close to the object/stranger. The movement of the mouse was tracked and recorded by an overhead video camera and analyzed by Smart Version 2.5 (Panlab-Harvard Apparatus).

#### Y-maze test

The Y-shaped maze was made of black Plexiglas with three arms (40 cm × 4 cm × 13 cm). For the test, animals were placed in the end of one arm and allowed to move freely through the maze for 7 min in dim light. An entry was defined as placing all four paws into an arm. The observer was blinded to the animal genotype. The percentage of spontaneous alterations was calculated as the ratio of the number of successful alterations to the number of total alterations minus 2.

#### Novel Object Recognition Test

The test was performed as described previously^33,34^. Before the task, habituation to a plastic chamber (30 × 30 × 30 cm) was conducted for 10 min over 2 consecutive days. After 2 days of habituation, the mice were presented with two identical objects and allowed to explore freely for 7 min for familiarization (acquisition trial). In the testing trial performed 24 h later, a novel object was substituted for one of the two objects (the familiar objects) and the mice were scored for recognition over a 7 min period. Object recognition was defined as spending time oriented toward the object at a distance of 1 cm or less, touching the object or sniffing with their nose. All of the objects were cleaned with 70% ethanol after each session to remove any odor cues. Exploration times were recorded manually and a discrimination index was calculated as: [exploration time with novel object − exploration time with familiar object]/[total exploration time].

### Drug preparation and *in vivo* injections

Thalidomide (Tocris Bioscience, Minneapolis, MN, USA) was dissolved to 25 mM in dimethylsulfoxide (DMSO) and then diluted 1:10 in saline for injection. Paxilline (Tocris Bioscience) was dissolved to 10 mM in DMSO and then diluted 1:2,000 in saline for injection. Thalidomide (30 mg/kg) or vehicle (10% (v/v) DMSO) was injected intraperitoneally of 24 h before open field test, tail suspension test, forced swimming test, elevated plus maze test, or the three-chamber social interaction tests. In the passive avoidance test and novel object recognition tests, the same dose of thalidomide was injected two times before the tests. Paxilline (3 μg/kg) or vehicle (0.05% (v/v) DMSO) was injected intraperitoneally 3 h before the tests.

### Slice preparation

Transverse hippocampal slices (300 µm) were prepared from 3- to 5-week-old mice (male only) as described previously^35^. Briefly, mice were anesthetized with isoflurane. After decapitation, the brains were rapidly removed and then submerged in ice-cold, oxygenated (95% O_2_ and 5% CO_2_), low-Ca^2+^/high-Mg^2+^ dissection buffer containing 5 mM KCl, 1.23 mM NaH_2_PO_4_, 26 mM NaHCO_3_, 10 mM dextrose, 0.5 mM CaCl_2_, 10 mM MgCl_2_, and 212.7 mM sucrose. Hippocampal tissues from both hemispheres were sectioned transversely using a vibratome (VT1000P or VT1200S; Leica Biosystems, Germany). The slices were then transferred to a recovery chamber containing oxygenated (95% O_2_ and 5% CO_2_) artificial cerebrospinal fluid (ACSF) containing 124 mM NaCl, 5 mM KCl, 1.23 mM NaH_2_PO_4_, 2.5 mM CaCl_2_, 1.5 mM MgCl_2_, 26 mM NaHCO_3_, and 10 mM dextrose and incubated at 28–30 °C for at least 1 h before recording. Experiments with thalidomide treatment were performed using hippocampal slices preincubated in ACSF containing thalidomide (100 μM) for at least 3 h.

### Whole-cell patch clamp recordings

Patch clamp recordings recording were performed as described previously^25^. After slice recovery or pre-incubation with thalidomide, slices were transferred to a submerged recording chamber and perfused continuously at 2 ml/min with oxygenated ACSF. Slices were equilibrated for 5 min prior to the recordings, and all of the experiments were performed at 23–25 °C. All recordings were performed in hippocampal CA1 pyramidal neurons identified by their size and morphology. Recordings were obtained using a Multiclamp 700B amplifier (Molecular Devices, Sunnyvale, CA, USA) under visual control with differential interference contrast illumination using an upright microscope (BX51WI; Olympus, Tokyo, Japan or Eclipse FN1; Nikon, Tokyo, Japan). Patch pipettes (4–6 MΩ) were filled with 135 mM K-gluconate, 8 mM NaCl, 10 mM HEPES, 2 mM ATP-Na, 0.2 mM GTP-Na (for calcium-activated potassium current experiments), 130 mM Cs-MeSO_4_, 0.5 mM EGTA, 5 mM TEA-Cl, 8 mM NaCl, 10 mM HEPES, 1 mM QX-314, 4 mM ATP-Mg, 0.4 mM GTP-Na, 10 mM phosphocreatine-Na_2_, 0.1 mM spermine (for paired pulse ratio of EPSC experiments) and maintained at pH 7.4 and 280–290 mOsm. The extracellular recording solution consisted of ACSF supplemented with picrotoxin (100 μM) for the EPSC experiment and tetrodotoxin (1 μM) for measurement of calcium-activated potassium currents. Evoked synaptic responses were elicited by SC stimulation (0.2 ms current pulses) using a concentric bipolar electrode placed 200–300 μm in front of the postsynaptic pyramidal cells. I_K(Ca)_ was measured as a peak amplitude of an outward tail current after brief (120 msec) depolarizing voltage steps from −50 mV to 0 mV^25,36^. Only cells with an access resistance < 20 MΩ and an input resistance > 100 MΩ were studied. The cells were discarded if the input or the access resistance changed by more than 20%. Data were acquired and analyzed using pClamp 10.2 (Molecular Devices). Signals were filtered at 2 kHz and digitized at 10 kHz with Digidata 1440 A or 1550B (Molecular Devices).

### Surface protein biotinylation and Western blot analysis

Biotinylation of proteins in the plasma membrane was performed as described previously^25^. Thalidomide was injected intraperitoneally into mice 48 h and 24 h before the slices preparation. Hippocampal slices were prepared from 8- to 16-week-old vehicle and thalidomide-treated mice using the same method of slice preparation as for electrophysiology. The slices were incubated for 40 min at 31 °C in oxygenated ACSF containing vehicle or thalidomide (100 μM). To biotinylate the cell surface proteins, slices were treated with sulfo-NHS-SS-biotin (Thermo Scientific, 21328, Waltham, MA, USA) in ACSF and incubated on ice for 45 min. After washing with ACSF, the biotin was quenched with 100 mM glycine in ACSF at 4 °C for 25 min. The quenched slices were lysed with RIPA buffer (50 mM Tris, pH 7.5, 0.1% SDS, 1% Triton X-100, 150 mM NaCl, 0.5 sodium deoxycholate and 2 mM EDTA) containing protease inhibitor cocktail (Sigma-Aldrich, 11836153001, St. Louise, MO, USA), and incubated at 4 °C rotating for 1 hr. The lysates were centrifuged at 15,000 × g for 15 min at 4 °C and supernatant was collected. Protein concentrations were determined via the BCA Protein Assay Kit (Thermo Scientific, 23225). To isolate biotinylated proteins, 200 μg of each sample was added to streptavidin-agarose beads (Thermo Scientific, 29200) and incubated at 4 °C rotating overnight. The streptavidin-agarose beads were washed with the lysis buffer and eluted with 2x laemmli sample buffer. Surface (biotin-labeled) and input (25% of total, 50 μg) proteins were separated on 10% SDS-PAGE gel. Separated proteins were then transferred onto PVDF membranes, blocked with 5% skim milk in TBS-T for 1 h at room temperature, and incubated overnight with BK channel antibodies (BD biosciences, 611249, 1:500, San Jose, CA, USA) in blocking buffer. Membranes were washed with TBS-T and then incubated with horseradish peroxidase (HRP)-conjugated secondary antibodies for 1 h at room temperature. HRP was detected using Super Signal West Pico Chemiluminescent substrate (Thermo Scientific, 34080) and a Bio-Image Analyzer (Bio-Rad ChemiDoc MP; Bio-Rad, Hercules, CA, USA).

### Statistical analysis

Data analyses and graphical displays were conducted with Prism 5.03 (GraphPad Software, La Jolla, CA, USA). All displayed values represent the mean ± SEM. Significant differences between groups were determined using unpaired Student’s t-tests, and multiple comparisons were performed using two-way ANOVA.
